# Dosimetric Impact of a Tumor Treating Fields Device for Glioblastoma Patients Undergoing Simultaneous Radiation Therapy

**DOI:** 10.3389/fonc.2018.00051

**Published:** 2018-03-13

**Authors:** Taoran Li, Gaurav Shukla, Cheng Peng, Virginia Lockamy, Haisong Liu, Wenyin Shi

**Affiliations:** ^1^Department of Radiation Oncology, Thomas Jefferson University, Philadelphia, PA, United States; ^2^Department of Radiation Oncology, University of Pennsylvania, Philadelphia, PA, United States

**Keywords:** glioblastoma multiforme, radiation therapy, tumor-treating fields, dose calculation, skin dose

## Abstract

**Purpose:**

A recent randomized phase III clinical trial in patients with glioblastoma demonstrated the efficacy of tumor treating fields (TTFields), in which alternating electric fields are applied via transducer arrays to a patient’s scalp. This treatment, when added to standard of care therapy, was shown to increase overall survival from 16 to 20.9 months. These results have generated significant interest in incorporating the use of TTFields during postoperative concurrent chemoradiation. However, the dosimetric impact of high-density electrodes on the scalp, within the radiation field, is unknown.

**Methods:**

The dosimetric impact of TTFields electrodes in the radiation field was quantified in two ways: (1) dose calculated in a treatment planning system and (2) physical measurements of surface and deep doses. In the dose calculation comparison, a volumetric-modulated-arc-therapy (VMAT) radiation plan was developed on a CT scan without electrodes and then recalculated with electrodes. For physical measurements, the surface dose underneath TTFields electrodes were measured using a parallel plate ionization chamber and compared to measurements without electrodes for various incident beam angles and for 12 VMAT arc deliveries. Deep dose measurements were conducted for five VMAT plans using Scandidos Delta4 diode array: measured doses on two orthogonal diode arrays were compared.

**Results:**

In the treatment planning system, the presence of the TTFields device caused mean reduction of PTV dose of 0.5–1%, and a mean increase in scalp dose of 0.5–1 Gy. Physical measurement showed increases of surface dose directly underneath by 30–110% for open fields with varying beam angles and by 70–160% for VMAT deliveries. Deep dose measurement by diode array showed dose decrease of 1–2% in most areas shadowed by the electrodes (max decrease 2.54%).

**Conclusion:**

The skin dose in patients being treating with cranial irradiation for glioblastoma may increase substantially (130–260%) with the addition of concurrent TTFields electrodes on the scalp. However, the impact of dose attenuation by the electrodes on deep dose during VMAT treatment is of much smaller, but measureable, magnitude (1–2%). Clinical trials exploring concurrent TTFields with cranial irradiation for glioblastoma may utilize scalp-sparing techniques to mitigate any potential increase in skin toxicity.

## Introduction

Glioblastoma remains the most common primary brain malignancy, with more than 13,000 patients diagnosed in 2017 in the United States (1). The median overall survival for patients diagnosed with glioblastoma is dismal, ranging from 15 to 16 months in prospective randomized studies performed in the first decade of this century (2, 3). This median survival resulted from the introduction of temozolomide, an alkylating chemotherapy given concurrently and adjuvantly with radiation therapy (RT) after maximal safe neurosurgical resection; prior to the use of temozolomide, clinical trials routinely demonstrated a median survival of approximately 12 months. The poor prognosis of this disease has motivated clinical trials of radiation dose escalation, novel drug therapies, and other unconventional approaches. The most successful of these approaches has been the introduction of tumor treating fields (TTFields), in which alternating electric fields are applied *via* transducer arrays to a patient’s scalp. This technique has been demonstrated to have anti-mitotic activity in tumor cells and showed promise in patients with recurrent disease (4–7). The technique was applied in patients immediately after the conclusion of adjuvant chemoradiation in a randomized phase III study, which showed increased overall survival from 16 to 20.9 months (8).

This remarkable improvement has generated significant interest in the use of TTFields earlier in the treatment course—that is, during the course of postoperative concurrent chemoradiation—in an attempt to evaluate the potential synergistic effect of multimodality therapy. However, the delivery of TTFields requires the placement of metallic transducer electrodes of high density onto the scalp of patients, directly into the radiation field. The dosimetric impact of an array of electrodes placed directly in the radiation field is not precisely known, and without this knowledge, estimation of the potential for toxicity with combined therapy is more difficult.

High-density objects on the surface of the patients receiving RT can have non-negligible and competing effects on the dose distribution in the patient’s body. On the one hand, the metal acts like additional buildup material, generating additional electrons *via* the photoelectric effect or Compton process. Clinically, this could translate into increased skin dose below the TTFields electrodes.

On the other hand, high-density materials can also act as an attenuator, reducing the dose to deeper tissues while simultaneously hardening the radiation beam *via* preferential attenuation of low energy photons. Clinically, this may impact the percentage depth dose curve and potentially decrease the target volume dose.

The present work is thus motivated by these physical principles, as well as the improvement in clinical outcomes with TTFields. We investigate both the buildup and attenuation/filtering effects of the TTFields electrode array on a scalp phantom, using both advanced dose calculation algorithms in the treatment planning environment and physical measurements of electrodes impact on surface and deep dose. We hypothesize that quantification of the dosimetric impact of the electrodes may facilitate the development and execution of clinical trials evaluating combination chemoradiation and TTFields for patients with newly diagnosed glioblastoma.

## Materials and Methods

### Planning Study

The planning study was done on the Anderson RANDO phantom. A CT scan of the phantom was acquired, with and without the TTFields device. Target volumes and organs-at-risk contours from 10 patients previously treated for glioblastoma were used to generate clinical contours on the RANDO phantom. The target and normal tissues from these cases were transferred to the RANDO phantom and then modified by a radiation oncologist to be as realistic as possible.

These phantom patients were each planned using volumetric-modulated-arc-therapy (VMAT) in Varian Eclipse^®^ treatment planning system. Planning techniques were duplicated from the original clinical treatment plans for the number of arcs and the arc angle ranges. The metal electrodes from the TTFields treatment devices were contoured on the CT scans and assigned a fixed density equal to the highest allowable value in each treatment planning system, which is around the density of aluminum, to reduce the impact from metal artifacts on dose calculation and maintain consistent electrode positions between different phantom plans. The dose calculation algorithm was set to AcurosXB v11 for its higher accuracy on calculating dose around high-Z materials (9).

All treatment plans were optimized on the scan without electrodes, which is how patients would be scanned and simulated during an actual clinical treatment plan design process. The prescription dose for all plans in this study was 60 in 2 Gy per fraction. Normal tissue objectives followed RTOG 0825 protocol: brainstem Dmax < 60 Gy, optical chiasm Dmax < 56 Gy, optical nerves Dmax < 55 Gy, lenses Dmax < 7 Gy, scalp Dmean < 20 Gy, scalp D20cc < 40 Gy, and scalp D10cc < 50 Gy. Plans were normalized so that the 100% prescription line covers 95% of the PTV volume. While keeping all plan parameters the same, the dose distribution was then recalculated on the phantom with the metal electrodes to assess the impact from the electrodes on key dosimetric parameters.

Paired comparison between treatment plans with and without electrodes were performed first for PTV coverage using percentage of PTV receiving at least Rx dose. The scalp dose was also compared to assess the dose buildup effect caused by the presence of metal object close to the skin. All comparisons were tested using Wilcoxon signed rank test to determine statistical significance.

### Physical Measurements

To further evaluate the impact of skin and deep doses from the presence of the TTFields electrodes, physical measurements were also conducted.

Surface dose changes were measured using a parallel plate ionization chamber (10). The chamber was placed in the solid water phantom with 10 cm backscatter, and the surface of the chamber was matched with the surface of the phantom. To protect the chamber, a 0.87 mm acrylic cap was placed on the front window of the parallel plate chamber, making the effective point of measurement at 1 mm depth in tissue equivalent media. The TTFields electrodes were then placed on top of the acrylic cap to mimic placement on a patient’s scalp. The radius of active volume of the chamber was much less than the radius of the electrodes; therefore, the partial volume effect as a result of the electrode partially blocking the chamber was minimized. All measurements were first performed with 10 cm × 10 cm field size at 100 cm SSD. Charges were collected with and without TTFields electrodes for incident beam angles at −85, −75, 60, 30, 0, 30, 60, 75, and 85° relative to the axis perpendicular to the surface of the chamber. In addition, surface doses with and without TTFields electrodes were also compared using actual VMAT plans delivered to a 20 cm × 20 cm × 20 cm solid water phantom. Surface doses for VMAT plans were measured with above-mentioned parallel plate chambers mounted with a special inset on the surface of the solid water phantom, with the chamber’s measurement window flush to the solid water phantom surface to mimic the actual scatter condition from the patient. Other *in vivo* dosimetry devices such as thermal luminescence dosimeter (TLD) or optically stimulated luminescence dosimeter (OSLD) were not chosen because of unavailability (TLD) and larger measurement uncertainty (~5% for OSLD).

**Figure 1:**
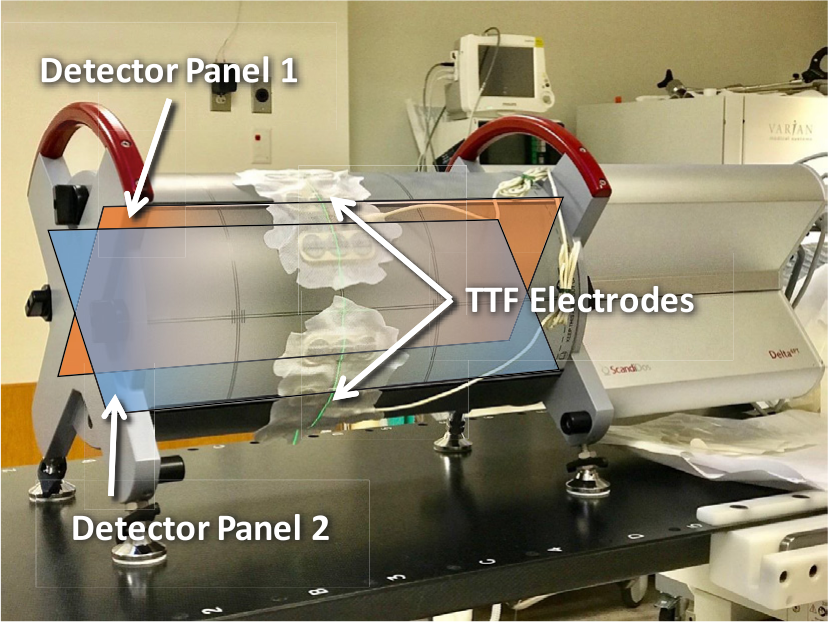
Measurements on deep dose impact from tumor treating fields (TTFields) electrodes conducted using the Delta4 volumetric-modulated-arc-therapy QA platform. Blue and amber overlays showing the position and directions of orthogonal detector panels 1 and 2.

In addition to surface dose, deep dose measurements were conducted using Delta4 device (Scandidos AB, Sweden) that are currently used clinically to verify all VMAT treatment in our clinic (11). As shown in Figure 1, this device has two planes of diode arrays that measures dose distribution within a cylindrical phantom. To mimic clinical treatment with TTFields, the electrodes were placed directly onto the surface of the phantom where beams would enter. The measurements were compared with and without electrodes. Histograms and mean dose deviation were used to assess deep dose differences caused by the TTFields electrodes.

## Results

### Plan Dosimetry Evaluation

**Figure 2:**
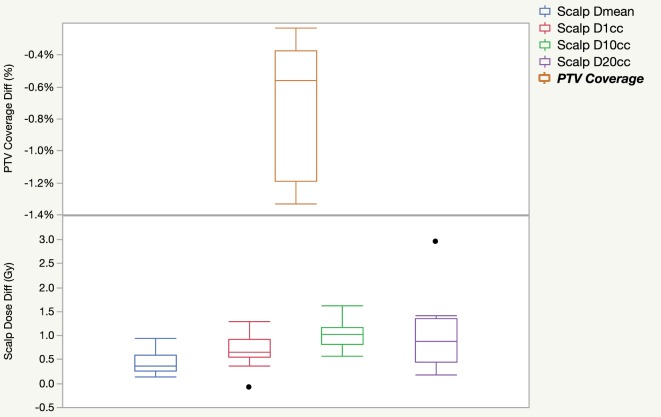
Dosimetric differences in PTV coverage and scalp dose as a result of including tumor treating field electrodes into dose calculation for 10 patients.

Figure 2 shows the difference in PTV coverage and scalp dose when comparing dose calculated with TTField electrodes in place against dose calculated without electrodes for 10 patients. In general, the calculated dose with the presence of electrodes decreased PTV coverage by 0.7 ± 0.4% in the treatment planning system. Dose to the scalp were slightly elevated when electrodes were taken into consideration during dose calculation. Mean scalp dose, as measured by the D1cc, D10cc, and D20cc were on average 0.5–1 Gy higher. Wilcoxon signed rank tests for all paired dosimetric parameters and the differences between with and without TTFields electrodes indicated were all statistically significant, with *p*-values ranging from 0.002 to 0.004.

**Figure 3:**
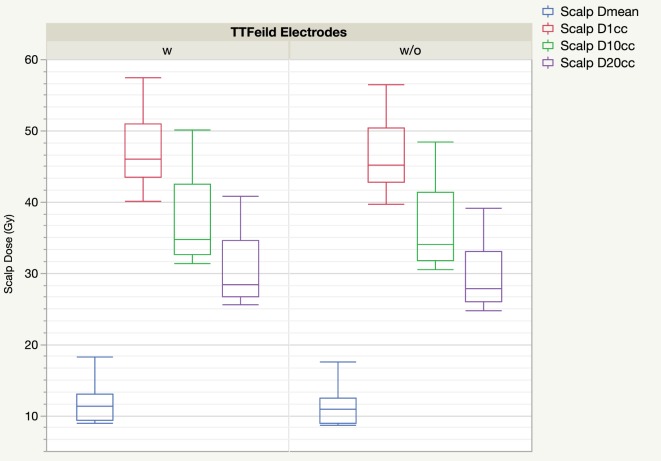
Boxplot of scalp DVH parameters from treatment planning system comparing with and without tumor treating field (TTField) electrodes.

Figure 3 shows the distribution of scalp mean dose, D1cc, D10cc, and D20cc of plans calculated with and without taking electrodes into consideration. The impact of the electrodes on the distribution of the scalp DVH parameter is generally small. Doses calculated with electrodes were numerically higher than without, but the paired differences were small although statistically significant.

### Physical Measurements—Surface Dose

**Figure 4:**
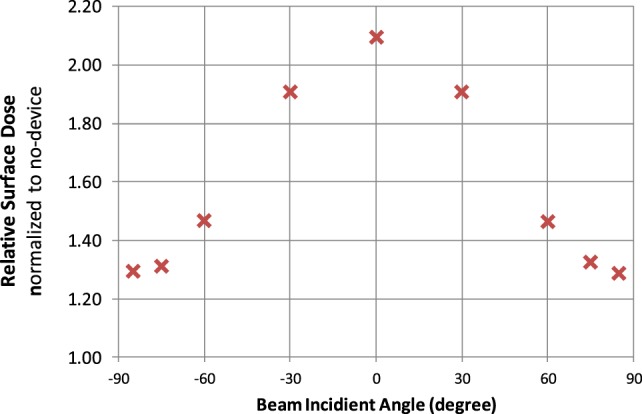
Surface dose increase factors plotted for different beam incident angles. Factors are normalized to the measurement taken at the same gantry angle without the electrodes.

Figure 4 shows the surface dose increase due to the presence of the electrodes. The surface dose measured at different gantry angles were compared to measurement done at the same condition but without the electrodes, and the results were plotted against gantry angles. It is evident that the presence of the TTFields electrode introduced additional buildup effect that increased surface dose directly underneath. This buildup effect was the most pronounced at gantry 0, i.e., beam being perpendicular to the surface, causing surface dose to be 2.1 times measurement without TTFields electrodes. At large angles, i.e., beam being near tangent to the surface, the buildup factor is smaller, but still increases the surface dose underneath the electrode by approximately 30%.

**Figure 5:**
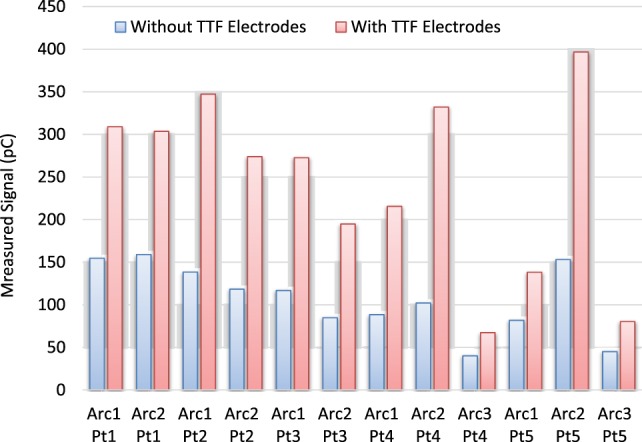
Surface dose comparison for volumetric-modulated-arc-therapy deliveries for 12 arcs and 5 patients. On average, the ratio of surface dose between measurement with tumor treating field (TTFields) electrodes and the one without is 2.23.

Figure 5 shows the surface dose measurement comparison for five VMAT plans (12 VMAT arcs) with and without TTFields electrodes. In all cases, the presence of TTFields electrodes significantly increased the surface dose measured by parallel plate chamber, by a mean ratio of 2.2 (range, 1.7–2.6), which was a larger effect than was measured in the open beam condition.

### Physical Measurements—Deep Dose

**Figure 6:**
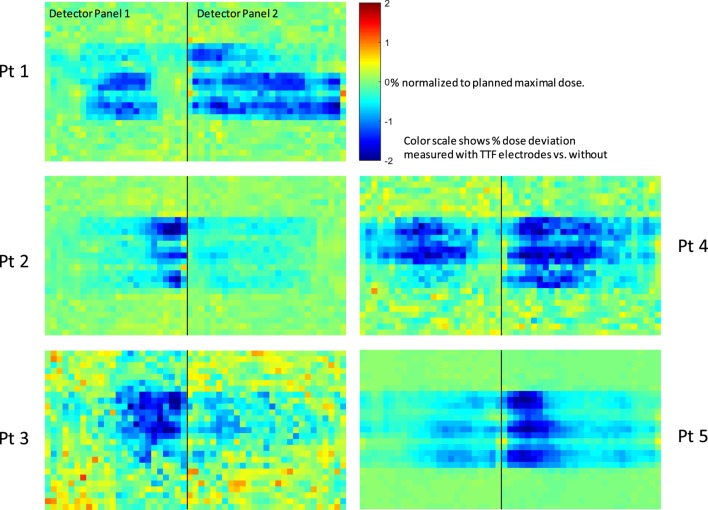
Deep dose measurement conducted using Delta4™ quality assurance device. Black line separates the two measurement planes orthogonally arranged in the cylindrical phantom. Pixels are color coded according to the dose difference relative to the maximal dose in the plan. The scales are from −2% (deep blue) to +2% (deep red).

Deep dose measurements for all five patients’ plans were shown in Figure 6. Regions around the center of the panels showed negative dose deviations, which corresponded to the locations that was shadowed by the TTFields electrodes. The pattern of the cold regions align well with the distribution of the individual electrodes on the TTFields device. Most regions affected by TTFields electrodes showed a dose decrease of 1–2%. Because the radius of the phantom is similar to that of an adult’s head, the impact on deep dose in a clinical scenario where patient receives treatment with TTFields electrodes is likely to be in the similar range.

## Discussion

Tumor treating fields has been gaining popularity in recent years as a treatment option for glioblastoma after completion of standard postoperative chemoradiation. In the clinical trials showing efficacy of TTFields, patients were prescribed to wear the device for greater than 18 h daily; this is not always feasible for patients. Utilizing the device in the setting of 6 weeks of daily radiation treatments may increase the logistical challenges to achieving high levels of compliance. From a clinical trial design standpoint, removing and replacing the electrodes on a daily basis to accommodate radiation may confound the outcome and increase skin irritation, since the electrodes are typically exchanged every 2–3 days. As such, before embarking on a trial to evaluate the possible synergy of these treatment modalities, it is necessary to quantify the impact of these high-density electrodes on the dosimetry of radiation treatment. The present work provides radiation oncologists and physicists some quantitative guidelines on how to safely design treatment plans for the delivery of concurrent therapy.

The results demonstrate that the presence of TTFields electrodes has minimal impact on the deep dose to the planning target volume, which suggests that tumor dose would be unlikely to be compromised due to shadowing from the electrodes. Importantly, the physical deep dose changes measured in the phantom agreed with the dosimetric changes, which is reassuring.

This dosimetric and physical dose measurement agreement was not seen in the quantification of skin dose. While no significant increase was observed in the treatment planning system using commonly evaluated DVH parameters such as scalp D1cc, physical measurements of surface dose increased when the electrodes were adhered to the surface by a factor of 1.3–2.6 in open field and VMAT deliveries. This result shows that additional caution must be taken into account for skin dose increase, which might not be evident in the DVH evaluation process. Scalp sparing could be improved by using a 3 mm contraction ring from the skin surface with additional penalizing constraints during VMAT optimization. The detailed effectiveness and trade-offs for using this technique is currently under investigation.

The results also provided an important guideline for the design of radiation treatment plans for treating glioblastoma patients concurrently with the TTFields device. Due to the increased dose measured at the surface, some effort to spare the scalp may be clinically meaningful. Further, scalp dose constraints may need to be more conservative, in anticipation of the additional skin dose below the electrodes. While the toxicity rates will have to be measured prospectively, a conservative recommendation might be to limit skin dose during treatment planning between 30 and 50% of published dose constraints.

There are several limitations of the current study. First, only one dose calculation algorithm was used in the planning study. For other does calculation algorithms and treatment planning systems, the difference between calculated dose with and without TTFields electrodes could be different, especially for the surface dose. Treatment planning systems have long had difficulty with accurate estimation of surface dose. However, as dose calculation algorithms improve, the accuracy of calculated skin/scalp dose is likely to be closer to measurements and may provide a more accurate skin dose estimation during treatment planning.

Second, although a parallel plate chamber was selected to ensure that dose is only measured at a very small depth (~1 mm), the measurement is limited by the large area of parallel plate chamber in the direction parallel to the surface. This limitation can partially explain why VMAT deliveries had a higher surface dose increase than the largest increase found in open field delivery. The ionization chamber used in this study has an active collection volume with a diameter of ~1 cm, while VMAT deliveries consist of many small and rapidly changing fields and segments. For deliveries without TTFields electrodes on top of the chamber window, when the chamber volume is partially exposed to a VMAT segment, only charges generated in the partial volume are collected but averaged over the entire volume. This results in a partial volume effect, which leads to an under-response with VMAT deliveries. When TTFields electrodes were placed on top of the parallel plate chamber’s collection volume, they served to act as a buildup material. In the same small segment scenario described above, even though only a part of the chamber is exposed to primary photons, secondary electrons generated from the TTFields electrodes can still be scattered into the part of collection volume that is not exposed to the primary photon fluence. This effectively increases the parallel plate chamber’s collection efficiency under the partial volume condition, which compensates for the partial volume effect and reduces the detector’s under-response. When the ratios between measurement with and without TTFields electrodes were calculated, the smaller denominator as a result of more pronounced partial volume effect could make the calculated ratio larger than actual value measured with a true point dose measurement.

When measuring deep dose, the TTFields electrodes were arranged to be in the same plane as the beam’s central axis of rotation. This maximized the likelihood of the electrodes being in the beam path. Therefore, the deep dose impacts detected in the above measurements likely represents a “worst case scenario.” In an actual clinical setting with patients wearing TTFields electrodes, the arrangement of TTFields electrodes will be non-coplanar and more random due to self-replacement by the patients, and the dosimetric impact is likely to be less than what was observed in the present work.

It is also important to note that the TTFields electrodes patches are disposable and regularly changed by patients. As the location of the electrodes will vary over the duration of RT, an effective averaging of the skin dose hot spots will be observed, which may reduce the maximal skin doses seen in any given region of the scalp. However, due to the uncertainty of this “hot spot averaging,” and in the absence of prospective data in patients, the authors recommend conservative scalp dose guidelines in clinical trials, with utilization of methods to reduce scalp dose as much as possible.

## Conclusion

In this study, the impact from TTField electrodes on the dosimetry of VMAT treatment plans for patients with glioblastoma was quantitatively evaluated in two ways: (1) in the treatment planning system using advanced dose calculation algorithm and (2) using physical measurements on surface and deep doses. In general, the presence of electrodes decreases deep dose by ~1–2% confirmed by both treatment planning dose calculation and physical measurements. Skin dose underneath the electrodes at ~1 mm depth were measured to be 1.3 – 2.6 times the dose measured without the electrodes, representing an increase by 30% to 160%; this effect was not evident from evaluation of scalp DVH parameters in the treatment planning system. The present work provides evidence that radiation doses to planning target volumes is not significantly changed due to the presence of TTFields electrodes, although the impact on skin dose was notable. As clinical trials to evaluate the potential synergy between TTFields and cranial irradiation are developed, care should be taken during treatment planning to reduce maximum scalp and skin doses well below published guidelines to reduce the risk of potential toxicity.

## Ethics Statement

The retrospective study was based on data with IRB approval.

## Author Contributions

TL contributed to the study design, data acquisition, data analysis, and led manuscript writing. GS contributed to the clinical aspect of study design and manuscript writing. CP and VL contributed to treatment plan generation. HL contributed to study design and data collection. WS is the PI of this project and provided overall guidance.

## Conflict of Interest Statement

The authors declare that the research was conducted in the absence of any commercial or financial relationships that could be construed as a potential conflict of interest.
